# Solvothermal Synthesis of a Novel Calcium Metal-Organic Framework: High Temperature and Electrochemical Behaviour

**DOI:** 10.3390/molecules26227048

**Published:** 2021-11-22

**Authors:** Russell M. Main, David B. Cordes, Aamod V. Desai, Alexandra M. Z. Slawin, Paul Wheatley, A. Robert Armstrong, Russell E. Morris

**Affiliations:** 1 EaStCHEM School of Chemistry, Purdie Building, North Haugh, St Andrews KY16 9ST, UK; rmm29@st-andrews.ac.uk (R.M.M.); dbc21@st-andrews.ac.uk (D.B.C.); avd6@st-andrews.ac.uk (A.V.D.); amzs@st-andrews.ac.uk (A.M.Z.S.); psw@st-andrews.ac.uk (P.W.); ara@st-andrews.ac.uk (A.R.A.); 2 Harwell Science and Innovation Campus, The Faraday Institution, Quad One, Didcot OX11 0RA, UK

**Keywords:** MOF, calcium MOF, electrochemistry, scXRD, VTXRD, bioMOF

## Abstract

The rapid growth in the field of metal-organic frameworks (MOFs) over recent years has highlighted their high potential in a variety of applications. For biological and environmental applications MOFs with low toxicity are vitally important to avoid any harmful effects. For this reason, Ca-based MOFs are highly desirable owing to their low cost and high biocompatibility. Useful Ca MOFs are still rare owing to the ionic character and large size of the Ca^2+^ ion tending to produce dense phases. Presented here is a novel Ca-based MOF containing 2,3-dihyrdoxyterephthalate (2,3-dhtp) linkers Ca(2,3-dhtp)(H_2_O) (SIMOF-4). The material undergoes a phase transformation on heating, which can be followed by variable temperature powder X-ray diffraction. The structure of the high temperature form was obtained using single-crystal X-ray diffraction. The electrochemical properties of SIMOF-4 were also investigated for use in a Na ion battery.

## 1. Introduction

Metal-organic frameworks (MOFs) are a rapidly growing field in materials chemistry [1]. They are formed from metal ions or oxoclusters, called secondary building units (SBUs), connected by polydentate organic molecules creating interconnected 3D networks [1]. The vast array of metal ions and organic linkers available means that a huge variety of structures with a wide range of topologies have already been discovered, and the designability and tunability of MOFs have been much discussed [2]. By definition [3] MOFs must have potential void space within the framework and MOFs have been discovered with internal surface areas up to around 10,000 m^2^/g [4]. The mix of organic and metallic components can provide a variety of novel functionalities [5,6,7,8,9,10,11,12,13,14,15,16,17,18,19,20,21], and while many applications for MOFs rely on their large porosities, recently more advanced MOFs have been developed that can respond to external stimuli (heat, pressure, light etc.,) [5] as well as MOFs that are stable in aggressive conditions [6]. Gas adsorption is one of the most well studied areas, with adsorption of fuels such as H_2_ [7] and CH_4_ [8], as well as carbon capture and storage [9]. Filtrations/separations are also possible [10], from improvements to industrial monomer refinements [11] to water and air purifications [12,13]. Other applications include, but are not limited to: catalysis [14], sensing [15] and anodes for batteries [16].

Of great interest to us is the use of MOFs in biological and environmental applications [17,18], particularly their use as drug carriers [19] and for the storage and release of biologically active NO gas [20,21]. To be used in biological systems it is important that the MOFs are non-toxic [17]. This is particularly true of the metal ions used: transition metals such as Ni and Cr are commonly used in MOF chemistry [1], but these metals are highly toxic [22]. Ca-based MOFs are highly desirable for biological applications because of their low cost and high biocompatibility [23]. Ca is important for bone health and many other biological processes, on average making up 1.4 wt.% of the human body [24]. Several Ca MOFs have been reported, however the high coordination number and ionic character of the Ca^2+^ ion tend to produce dense phases or Ca layers separated by the linkers [25,26]. We have previously reported a Ca MOF for NO delivery for use in biomedical applications [23].

The use of Ca MOFs is not just limited to biology, some Ca MOFs have been reported that show potential as anodic materials in Na ion batteries [27]. The potential of Na ion batteries has been widely discussed, with the aim of using them on a large scale for energy storage [28].

The stability of MOFs is also an important consideration, as they are rarely the thermodynamic product of a reaction and are instead kinetically stable intermediates [29]. The structures of MOFs are susceptible to collapse due to the application of heat, varying pH and the removal of internal solvent [6]. Some MOFs can undergo phase transitions on heating caused by rearrangement to a more thermodynamically stable product and/or the removal of internal solvent molecules [30,31]. Typically, these phase transitions cause structural damage owing to large changes in unit cell volume, the phase change going through an amorphous intermediate [32] and/or capillary forces caused by loss of solvent applying large stresses to the structure [1]. This can make structure determination of high temperature desolvated phases by scXRD challenging [32], as single crystals are almost inevitably damaged by this process. Often only a partial structure solution is possible.

Reported here is a new calcium MOF, SIMOF-4, based on 2,3-dihyrdoxyterephthalate (2,3-dhtp) linkers, with a 3D interconnected framework. Its crystal structure has been solved by scXRD, it has been characterised using PXRD and TGA. A high temperature transition to a layered material has been observed and its structure is determined using SCXRD on a damaged crystal. The structure was then confirmed using PXRD. Finally, its preliminary suitability for use as a battery anode have been assessed by measuring its electrochemical properties.

## 2. Results and Discussion

### 2.1. Synthesis Route

The linker 2,3-dihydroxyterephthalic acid (2,3-dhtpH_2_) was synthesised from the carboxylation of catechol using the Kolbe-Schmidt method [33]. This involved heating dry catechol in a CO_2_ atmosphere in the presence of excess potassium bicarbonate. This produced the potassium salt of 2,3-dihyrdoxyterephthalate from which 2,3-dhtpH_2_ could be formed on acidification.

SIMOF-4 was synthesised using a solvothermal technique. The reactants were dissolved in solvent and heated in an autoclave to 120 °C for 3 days. Acetic acid was used as the modulator to aid crystal growth [1]. Adding *p*-xylene (*p*X) caused a reduction in yield but an increase in crystal size allowing for scXRD analysis to be completed. The nature of the effect of *p*X is still undetermined: it has been known to act as a templating agent [34], but in this case we suggest that it is only acting to hinder nucleation and therefore improve crystal size.

### 2.2. Description of Crystal Structure

**Figure 1:**
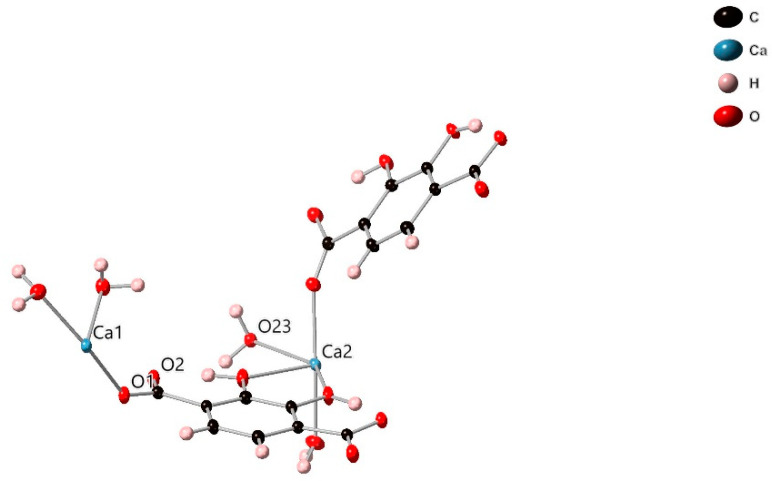
Thermal ellipsoid plot (50% probability ellipsoids) showing the asymmetric unit of SIMOF-4.

SIMOF-4 crystallises in the triclinic space group *P*$\bar{1}$. Its structure consists of a three-dimensional network based on a [Ca(2,3-dhtp)(H_2_O)]_2_ asymmetric unit (Figure 1), which contains two unique Ca environments.

**Figure 2:**
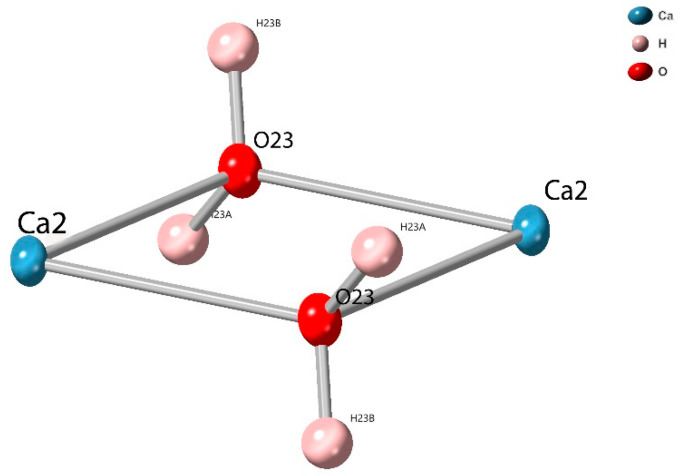
View of the Ca2 dimer of SIMOF-4 (50% probability ellipsoids).

**Figure 3:**
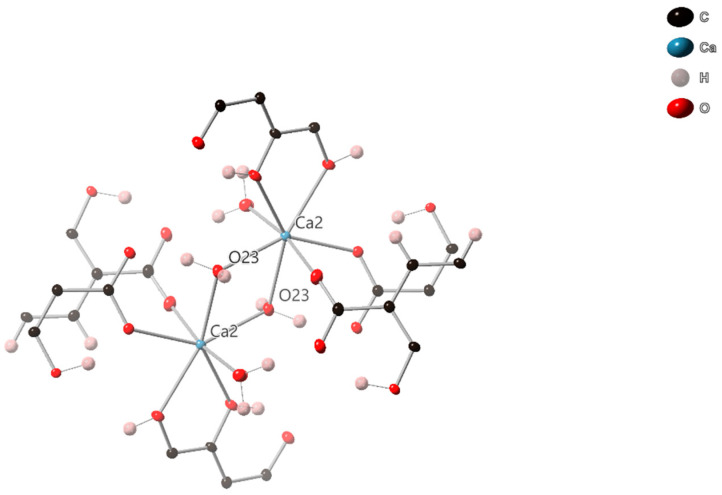
View of the bonding environment around the Ca2 dimer in SIMOF-4 (50% probability ellipsoids).

Ca2 is 7-coordinate with a distorted pentagonal bipyramidal geometry, and forms a dimer with a symmetry-related Ca via two bridging µ_2_ water molecules (O23) (Figure 2). The intermetallic distance is 4.0123(7) Å and the O-Ca-O angle is 71.80(5)°. The Ca2 is further coordinated to both phenolic groups of a µ_4_ dhtp, with a bite angle of 64.56(4)° and two µ_3_ dhtp groups through a carboxylic acid oxygen, a water molecule makes up the remaining coordination site (Figure 3). All Ca-O bonds are between 2.2678(15) Å and 2.5167(14) Å, all O-Ca-O angles are between 64.56(4)° and 170.36(5)°.

**Figure 4:**
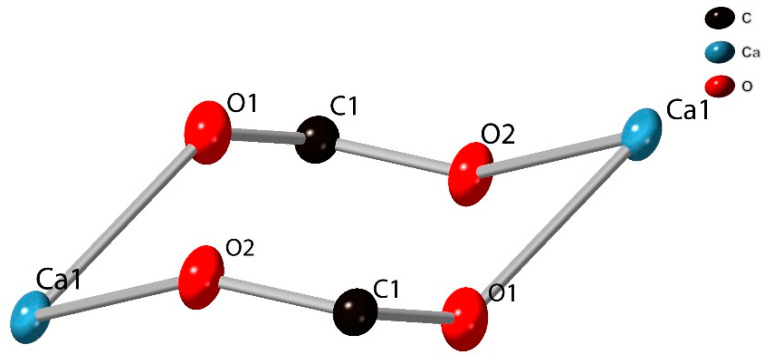
View of the Ca1 dimer unit of SIMOF-4 (50% probability ellipsoids).

**Figure 5:**
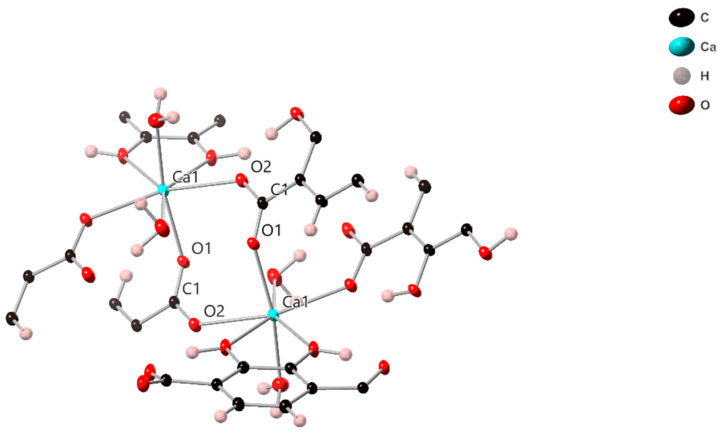
View of the bonding environment around the Ca1 dimer in SIMOF-4 (50% probability ellipsoids).

Ca1 is also 7 coordinates with a distorted pentagonal bipyramidal geometry, and forms a dimer with a symmetry-related Ca, in this case via two µ_4_ dhtp carboxylic acid groups (O1, C1, O2), (Figure 4). The intermetallic distance is 5.035(11) Å. The Ca1 is further coordinated to both phenolic groups of a µ_3_ dhtp, with a bite angle of 64.21(4)°, and two µ_4_ dhtp groups via carboxylic acid oxygens, a water molecule makes up the remaining coordination site (Figure 5). All Ca-O bonds are between 2.2917(15) Å and 2.4646(13) Å all O-Ca-O angles are between 64.21(4)° and 167.64(5)°.

**Figure 6:**
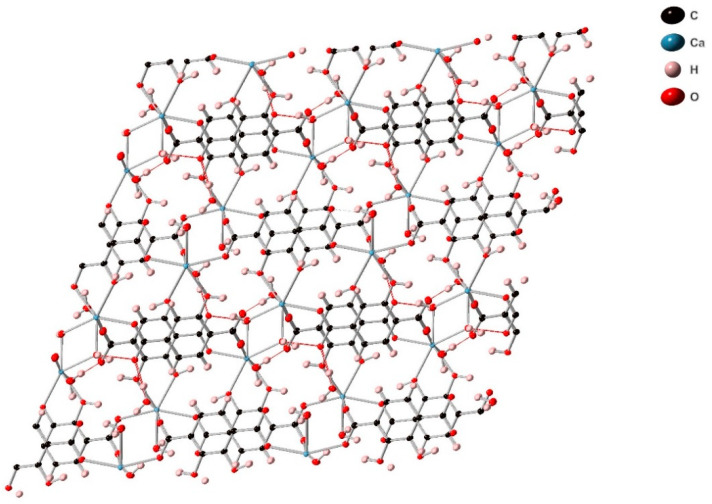
View of the 3D structure of SIMOF-4 (50% probability ellipsoids) as seen down the crystallographic *a*-axis, and showing hydrogen bonds (red).

This high connectivity produces an interconnected 3D network (Figure 6, Figures S1 and S2, Supplementary Materials). SIMOF-4 gains further stabilisation from hydrogen bonding, with each unbonded carboxylic oxygen hydrogen bonded to a water molecule with O···O separations between 2.6379(64) Å and 2.6947(67) Å. This structure appears non-porous. However, there are potential small voids occupied by bound water molecules. This indicates that removal of these water molecules could allow access of small molecules into the structure.

### 2.3. Characterisation

#### 2.3.1. Powder X-ray Diffraction

**Figure 7:**
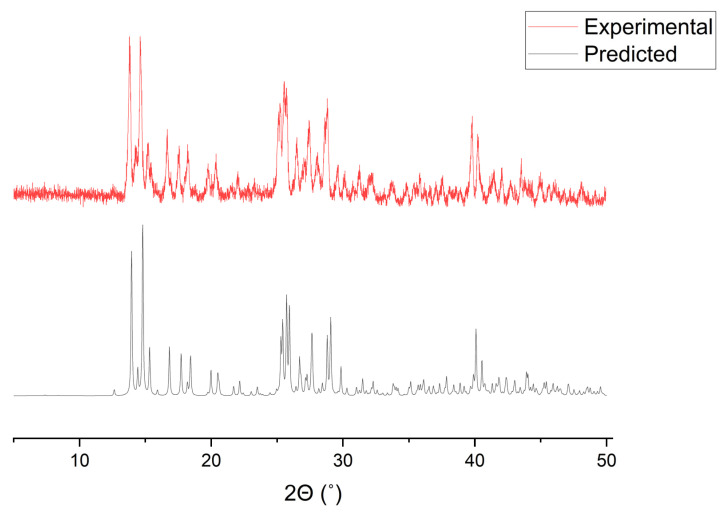
The simulated and experimental PXRD pattern of SIMOF-4 at room temperature.

The powder X-ray diffraction (PXRD) data pattern of SIMOF-4 is shown in Figure 7. The positions of the reflections are consistent with the theoretical pattern calculated from the single crystal X-ray structure.

#### 2.3.2. Thermo-Gravimetric Analysis

**Figure 8:**
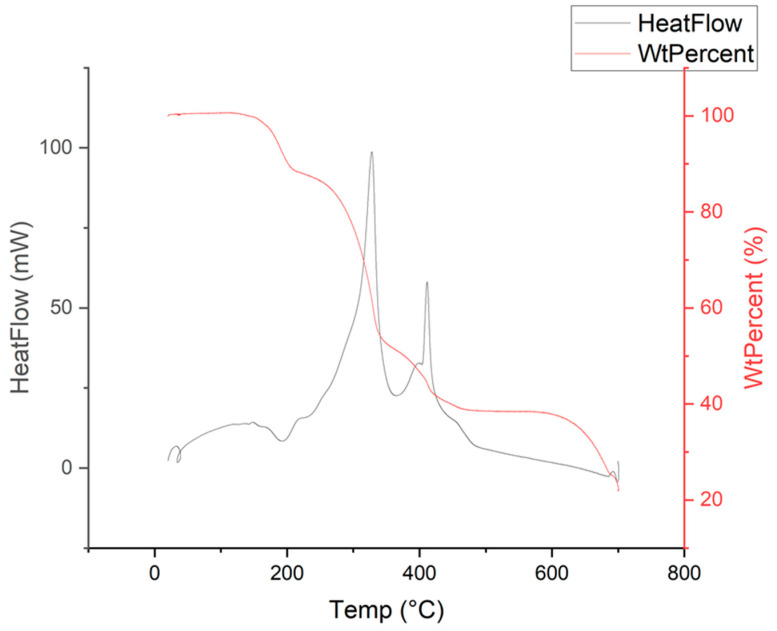
Thermo-gravimetric analysis of SIMOF-4, taken at a ramp rate of 5 °C/min.

Thermo-gravimetric analysis (TGA) shows multiple mass losses (Figure 8). The 12 wt.% mass loss between 135 and 215 °C, is endothermic and is consistent with loss of all water from the crystal structure. The two-step mass loss between 250 and 480 °C is exothermic and is consistent with degradation to CaCO_3_. This is backed up by PXRD analysis after a 480 °C heat treatment, which matches theoretical peaks for CaCO_3_ (Figure S3). The final mass loss at >600 °C is consistent with the decomposition to CaO as shown by Karunadasa et al. [35]. The lack of mass loss before 135 °C indicates that there is no solvent within the structure, apart from the water bound to the metals.

#### 2.3.3. Variable Temperature Behaviour

**Figure 9:**
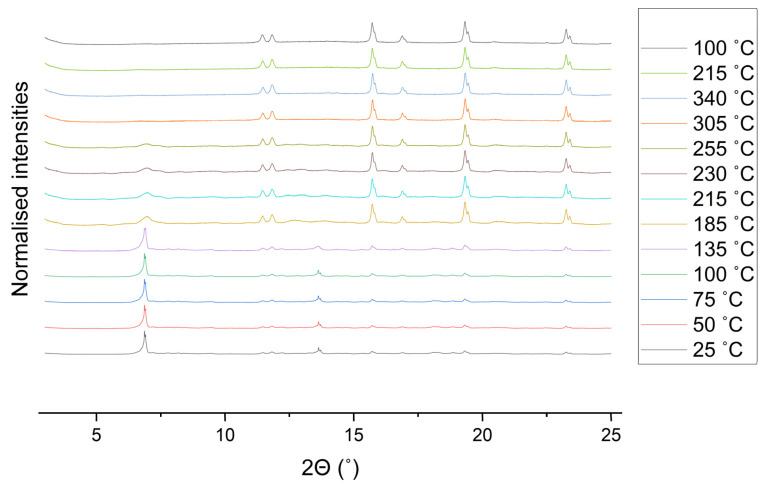
Main: normalised VTXRD patterns of SIMOF-4 taken on a corundum disc. All diffraction peaks in the patterns above 305 °C are from the corundum sample holder.

The water loss and first degradation step at 300 °C were explored further by use of variable temperature X-ray diffraction (VTXRD) (Figure 9). The VTXRD indicates the MOF is stable up to 100 °C, and that at 135 °C the pattern shows a subtle broadening indicating the start of a change in the structure, likely due to water loss. At 185 °C the pattern changes significantly: the major peak at 6.87° shifts to 6.95° and becomes much broader, the sharp peak at 13.62° is replaced by three broad peaks between 12.27 and 14.44°. This suggests that loss of water induces a change in the crystal structure, although the broadening of the peaks also suggests a reduction in crystallinity. Above 305 °C the only Bragg diffraction peaks visible come from the corundum sample holder indicating that the MOF has become amorphous, which shows that this peak in the DTA is destruction of the MOF. On cooling this amorphous structure remains. These results were confirmed by ex situ heat treatment and analysis (Figure S3).

#### 2.3.4. Single Crystal Structure of High Temperature Form

The phase transition at high temperature caused significant damage to the crystals; they underwent a change in morphology and became significantly less diffracting, this reduced their suitability for scXRD. This was true for all crystals we investigated. However, careful crystal selection and truncating the scXRD data did give diffraction that was good enough to solve, producing a plausible structure. This is not unusual in this type of science. As suggested by TGA, all water is lost, producing Ca(2,3-dhtp) (SIMOF-4-h), and this induces a significant change to the crystal structure. The unit cell remains triclinic but the unit cell parameters change, leading to a reduction in the unit cell volume by ≈18%. While we cannot infer any fine detail of the atomic arrangement and atomic displacement parameters, it is notable that this model does reproduce the experimental powder X-ray diffraction pattern well (Figure S4), and so some conclusions about the structure may be drawn from this model.

**Figure 10:**
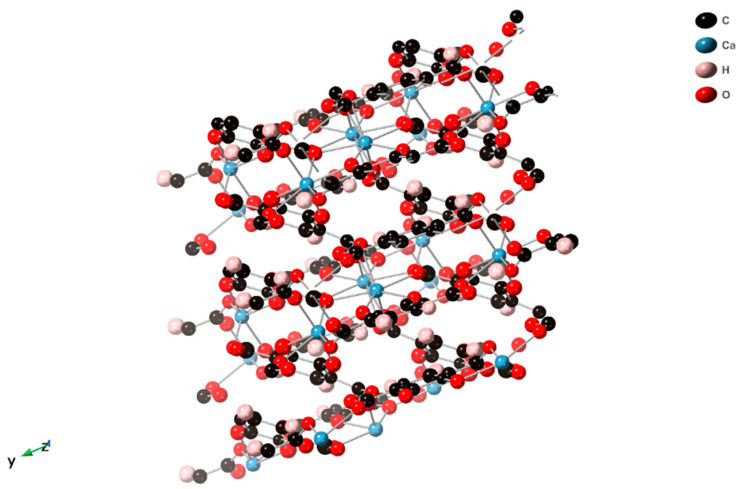
View of the crystal structure of SIMOF-4 (50% probability ellipsoids) after dehydration via heat treatment (down the crystallographic *c*-axis).

The phase change produces a dense interconnected structure that appears to show Ca and 2,3-dhtp layers in the *ab*-plane connected by further 2,3-dhtp linkers (Figure 10). The loss of water is detrimental to the stability of SIMOF-4 causing loss of crystallinity, as the structure changes to a layered phase. This collapse is most likely driven by loss of water from the Ca2 dimer units (Figure 2) causing structure rearrangements to maintain the 7 coordinate environment around the Ca ions.

**Figure 11:**
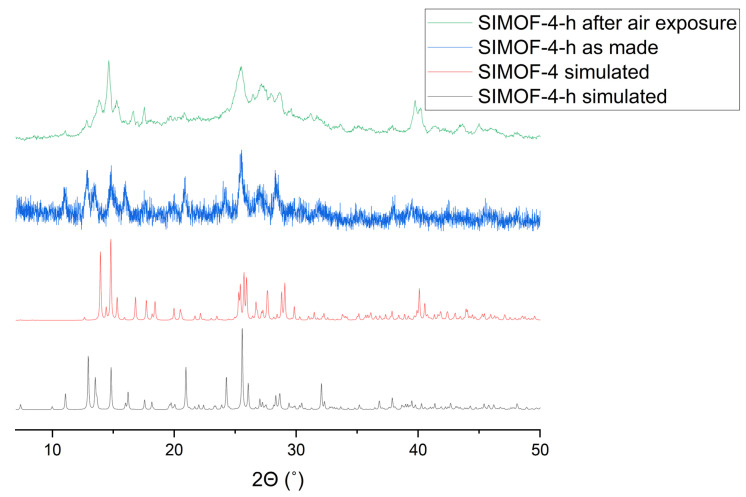
Simulated PXRD patterns of SIMOF-4 and SIMOF-4-h (high T form) and experimental patterns of SIMOF-4-h as synthesized and after prolonged exposure to air. All patterns obtained at room temperature.

SIMOF-4-h tended to degrade in aqueous conditions and so rehydration was initially unsuccessful. However, prolonged exposure to air, approximately 3 months, caused a transition back to the hydrated form, SIMOF-4 (Figure 11).

#### 2.3.5. Electrochemistry

Initial cyclic voltammetry (CV) was performed on SIMOF-4 between 0.01 and 2.5 V vs. Na^+^/Na at a scan rate of 0.05 mV s^−1^. For the first cycle a low-intensity reductive peak was observed at 1.95 V and another broad peak at ~0.15 V having a shoulder at ~0.56 V (Figure S6). No prominent oxidative peaks were observed, except a very broad profile between 1 and 1.7 V and another between 1.7 and 2.5 V. In the second cycle the reductive peak was shifted to ~1.48 V, while the remaining profile remained featureless. The shift continued in the 3rd cycle also along with a lowering of the peak height, which could be indicative of conversion to an amorphous phase [36] or structural changes, possibly to the dehydrated form above.

**Figure 12:**
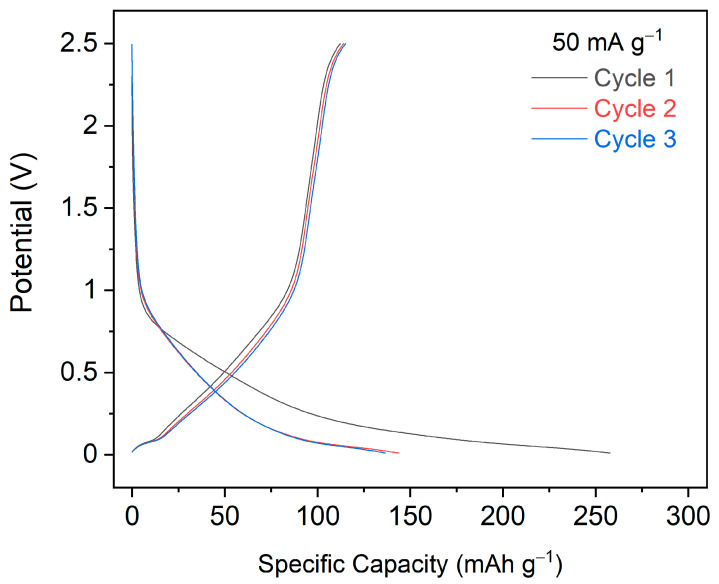
Galvanostatic charge/discharge curves for the first three cycles of SIMOF-4 recorded between 0.01 and 2.5 V at a current density of 50 mA g^−1^.

**Figure 13:**
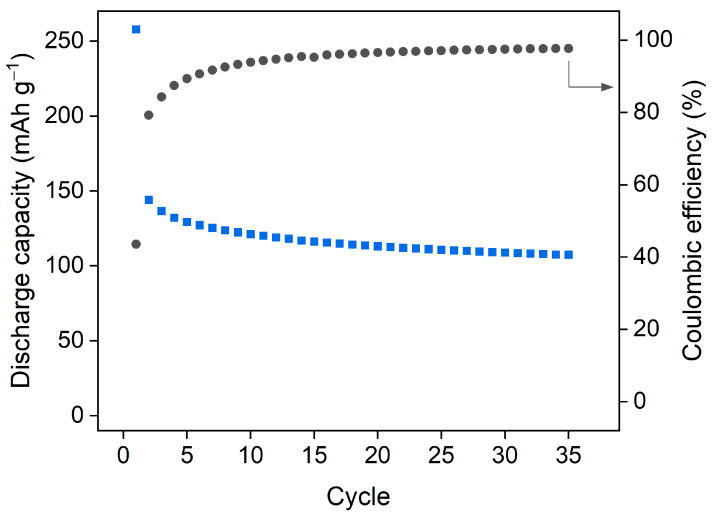
Discharge capacity and coulombic efficiency during galvanostatic cycling between 0.01 and 2.5 V at a current rate of 50 mA g^−1^.

Subsequently, galvanostatic charge/discharge cycling was carried out at a current density of 50 mA g^−1^ (Figure 12 and Figure 13). A high capacity of ~257 mAh g^−1^ was observed for the first discharge with a low coulombic efficiency (43%). This could be indicative of the contribution of SEI (solid-electrolyte interphase) layer formation [37] and changes to the structure or amorphization as anticipated from CV profiles. The load curves did not show long voltage plateaus and exhibited overlapping traces from the 3rd cycle onwards (Figure 12). The discharge capacity reduced to 144 mAh g^−1^ in the second cycle and had only a gradual fade and stabilised at ~110–120 mAh g^−1^ in the following cycles (Figure 13). This is a moderate discharge capacity [38] but one that does suggest some promise for this type of material.

## 3. Materials and Methods

### 3.1. General Remarks

All chemicals were purchased commercially and applied directly. PXRD was performed on a STOE STADIP diffractometer (Germany) using Cu (Kα1) radiation monochromated with a curved Ge (111) crystal. PXRD of SIMOF-4-h was collected with Mo (Kα1) radiation monochromated with a primary beam monochromator. VTXRD was performed on a PANalytical Empyrean spectrometer (UK/The Netherlands) using Mo (Kα1,2) radiation, the heating profile is provided in the supplementary information (Figure S5). TGA was performed on a Stanton Redcroft STA-780 simultaneous TG-DTA (UK) with a ramping rate of 5 °C/min. N_2_ adsorption isotherms were collected on a Micromeritics ASAP 2020 (USA), activation was performed at 25 °C, 100 °C, and 150 °C all under vacuum. NMR was performed on a Bruker AVII 400 instrument (Germany), with sample dissolved in DMSO-*d_6_*.

### 3.2. Preparation of 2,3-Dhtp

Oven-dried catechol (7.5 g, 68.1 mmol) and potassium carbonate (20.46 g, 204 mmol) were placed in an autoclave. The autoclave was flushed with a vacuum/N_2_ cycle three times and then charged with CO_2_ to a pressure of 10 bar. The vessel was heated to 230 °C incrementally and left overnight. The product was cooled and the solid crushed and suspended in water (300 mL). The liquid was separated via centrifugation at 6000 rpm, and HCl (25 mL) was added. The resulting precipitate was filtered, washed with water and ethanol and dried in an oven overnight to produce a pink powder of 2,3-dhtpH_2_. ^1^H-NMR: 7.27 (2H, s). ^13^C-NMR: 172.0 (1C, s), 151.2 (1C, s), 119.0 (1C, s), 117.2 (1C, s).

### 3.3. Preparation of SIMOF-4

Ca(NO_3_)_2_·4H_2_O (2.36 mg, 1 mmol) and 2,3-dhtpH_2_ (198 mg, 1 mmol) were dissolved via sonication in either 10 mL THF, 5 mL water and 5 mL ethanol for bulk product or 5 mL THF, 5 mL water, 5 mL ethanol and 5 mL *p*-xylene for single crystals. Acetic acid (5.25 mmol) was added, and the mixture was placed in an autogenous autoclave and heated to 120 °C for three days. The mixture was then cooled to room temperature and the product separated by filtration, washed with water and dried in an oven to produce grey crystals of the new Ca MOF (Yield: bulk 44%, single crystal 14%).

### 3.4. Preperation of SIMOF-4-h

Previously prepared SIMOF-4 was heated to 185 °C at 1 °C/min and the temperature was maintained for 3 h with exposure to air. The sample was then cooled to room temperature in an N_2_ environment producing matte grey crystals of SIMOF-4-h.

### 3.5. X-ray Crystallography

Selected crystals of SIMOF-4 were analysed using a Rigaku FR-X Ultrahigh Brilliance Microfocus RA generator/confocal optics with XtaLAB P200 diffractometer [Mo Kα radiation (λ = 0.71073 Å)]. SIMOF-4-h crystals were analysed using a Rigaku MM-007HF High Brilliance RA generator/confocal optics with XtaLAB P100 diffractometer [Cu Kα radiation (λ = 1.54187 Å)]. Data collection was performed using CrystalClear [39], data reduction and cell refinement were performed using CrysAlisPro [40]. The heat treated crystals showed extremely weak diffraction and data were truncated at 1.06 Å. Structure solution was performed with SHELXT-2018/2 [41] version 2018/2, refinement was performed with SHELXL-2018/3 [42]. In SIMOF-4, non-hydrogen atoms were refined anisotropically, and CH hydrogen atoms were refined using a riding model. Hydroxyl hydrogen atoms were located from the difference Fourier map and refined isotropically subject to a distance restraint. In SIMOF-4-h, calcium atoms were refined anisotropically, while other non-hydrogen atoms were refined isotropically, and CH hydrogen atoms were refined using a riding model. Hydroxyl hydrogen atoms were placed in calculated positions, selected on the basis on neighbouring atoms, and refined with a riding model. Due to the poor data-quality in SIMOF-4-h, the benzene rings of the 2,3-dhtp ligands were constrained to ideality. All calculations were performed using the Olex2 [43] interface. Deposition numbers 2113160 and 2117049 contain the supplementary crystallographic data for this paper.

Crystal data (SIMOF-4). C_8_H_8_CaO_8_, *M* = 272.22, triclinic, *a* = 7.1339(3), *b* = 11.5312(5), *c* = 13.1000(8) Å, *α* = 66.744(5), *β* = 80.811(4), *γ* = 80.834(4)°, *U* = 971.86(9) Å^3^, *T* = 93 K, space group *P*$\bar{1}$ (no. 2), *Z* = 4, 11,622 reflections measured, 4197 unique (*R*_int_ = 0.0215), which were used in all calculations. The final *R*_1_ [*I* > 2*σ*(*I*)] was 0.0403 and *wR*_2_ (all data) was 0.1186.

Crystal data (SIMOF-4-h). C_8_H_4_CaO_6_, *M* = 236.19, triclinic, *a* = 7.350(4), *b* = 9.540(6), *c* = 12.734(6) Å, *α* = 73.55(5), *β* = 73.92(5), *γ* = 71.84(5)°, *U* = 796.1(8) Å^3^, *T* = 173 K, space group *P*$\bar{1}$ (no. 2), *Z* = 4, 2055 reflections measured, 1300 unique (*R*_int_ = 0.0549), which were used in all calculations. The final *R*_1_ [*I* > 2*σ*(*I*)] was 0.1835 and *wR*_2_ (all data) was 0.4867.

### 3.6. Electrochemical Testing

The working electrodes for SIMOF-4 were prepared in a mixture of active compound (55%), conducting carbon—Super C65 (35%) and binder—CMC (carboxymethyl cellulose, 10%). A slurry was prepared in water and cast on an aluminium foil (Advent Research Materials, UK) by a doctor blade. The electrodes were subsequently punched (~12 mm diameter) upon air drying and dried overnight in a vacuum oven at 110 °C and transferred to an argon-filled glovebox. The average mass loading of the active material per electrode was 1.54 mg cm^−2^.

Electrochemical testing was done using coin cells (CR2325), that were assembled using sodium (Sigma-Aldrich) as the counter electrode and a glass fiber separator (Whatman GF/F). NaPF_6_ in EC:DEC (1:1, ethylene carbonate—EC, diethyl carbonate—DEC) was used as the electrolyte and the process was carried out in the glovebox with oxygen and water content <1 ppm. Electrochemical measurements were recorded at 30 °C on a Biologic BCS-805 modular battery testing system in a potential window of 0.01–2.5 V (vs. Na^+^/Na) and all data were analysed and processed using the BT-Lab software.

## 4. Conclusions

In this paper we have presented a new Ca MOF, SIMOF-4. The crystal structure of the as-made material has been determined using single-crystal X-ray diffraction, and its high temperature behaviour was studied using variable temperature PXRD and TGA. Despite significant damage to the single crystals on thermal treatment the structure of the high temperature phase was also determined. Initial electrochemistry experiments on the material’s suitability for use as an anode in sodium-ion batteries showed that SIMOF-4 has moderate discharge capacity. These results present two possible avenues for future work. First, improving the discharge capacity to improve stability and creating a non-toxic anode for Na-ion batteries that may be viable in this type of material. Second, the replacement of water within the pores of the MOF with small biologically active molecules such as NO may yield non-toxic materials of potential utility.

## Data Availability

CCDC-2113160 and 2117049 contains the supplementary crystallographic data for this paper. These data are provided free of charge by the joint Cambridge Crystallographic Data Centre and Fachinformationszentrum Karlsruhe Access Structures service www.ccdc.cam.ac.uk/structures. All other data can be obtained from the authors on request.

## Supplementary Materials

The following are available online. Figure S1: View of the 3D structure of SIMOF-4 (50% probability ellipsoids) as seen down the crystallographic *b*-axis, and showing hydrogen bonds. Figure S2: View of the 3D structure of SIMOF-4 (50% probability ellipsoids) as seen down the crystallographic *b*-axis, and showing hydrogen bonds. Figure S3: PXRD patterns of SIMOF-4 after different heat treatments, with simulated patterns of CaCO_3_ and SIMOF-4 for comparison. All obtained using Cu(Kα1) radiation. Figure S4: PXRD pattern of heat treated SIMOF-4 simulated from scXRD structure compared with the experimental pattern. Obtained using Mo(Kα1) radiation. Figure S5: Cyclic voltammogram for SIMOF-4 recorded at a scan rate of 0.05 mV s^−1^ between 0.01–2.5 V. Figure S6: N_2_ Adsorption isotherms of SIMOF-4 after three different activation protocols of 25 °C, 100 °C and 150 °C all under vacuum. Figure S7: Temperature time profile for the VTXRD experiment performed on SIMOF-4, showing programmed and measured temperature profiles.

## Sample Availability

Samples of the compounds are available from the authors on request.
