# Supplementary material for: Solvothermal Synthesis of a Novel Calcium Metal-Organic Framework: High Temperature and Electrochemical Behaviour
Source: Molecules. 2021 Nov 22;26(22):7048. doi: 10.3390/molecules26227048 (PMC8623775; doi:10.3390/molecules26227048)
Supplement: Supplementary file 1 [file molecules-26-07048-s001.zip › molecules-1456000-supplementary.pdf]

molecules

Solvothermal Synthesis of a Novel Calcium Metal-Organic Framework: High Temperature and Electrochemical Behaviour

Supplementary Material

Russell M. Main,[1] David B. Cordes,[1] Aamod V. Desai,[1,2] Alexandra M. Z. Slawin,[1] Paul Wheatley,[1] A. Robert Armstrong[1,2] and Russell E. Morris[1,2,*]

1 EaStCHEM School of Chemistry, Purdie Building, North Haugh, St Andrews KY16 9ST, UK
2 The Faraday Institution, Quad One, Harwell Science and Innovation Campus, Didcot, OX11 0RA, UK
* Correspondence: rem1@st-andrews.ac.uk

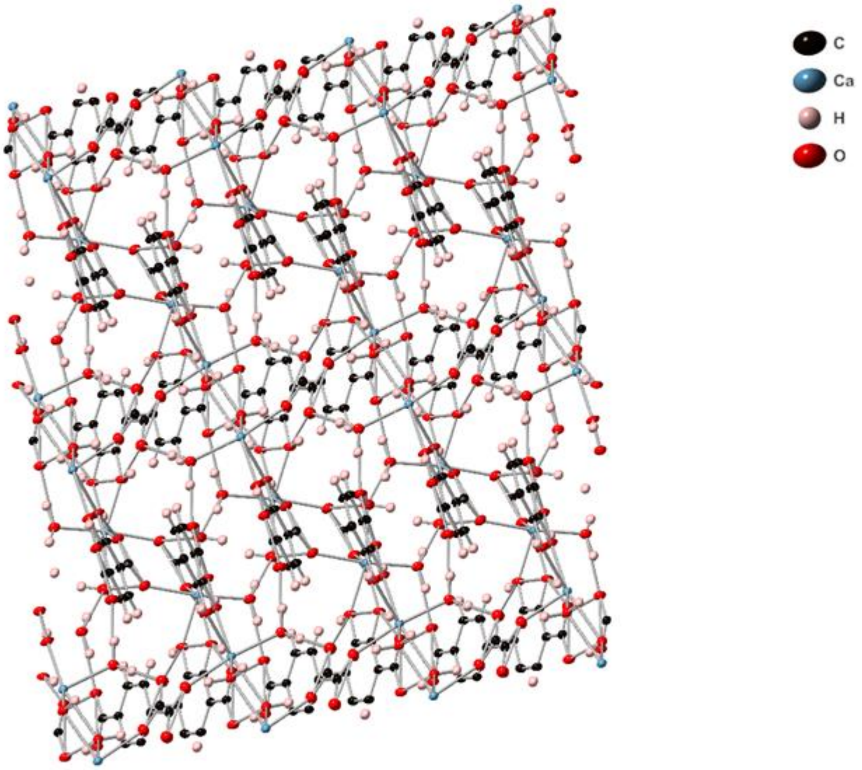

Figure S1: View of the 3D structure of SIMOF-4 (50 % probability ellipsoids) as seen down the crystallographic b-axis, and showing hydrogen bonds.

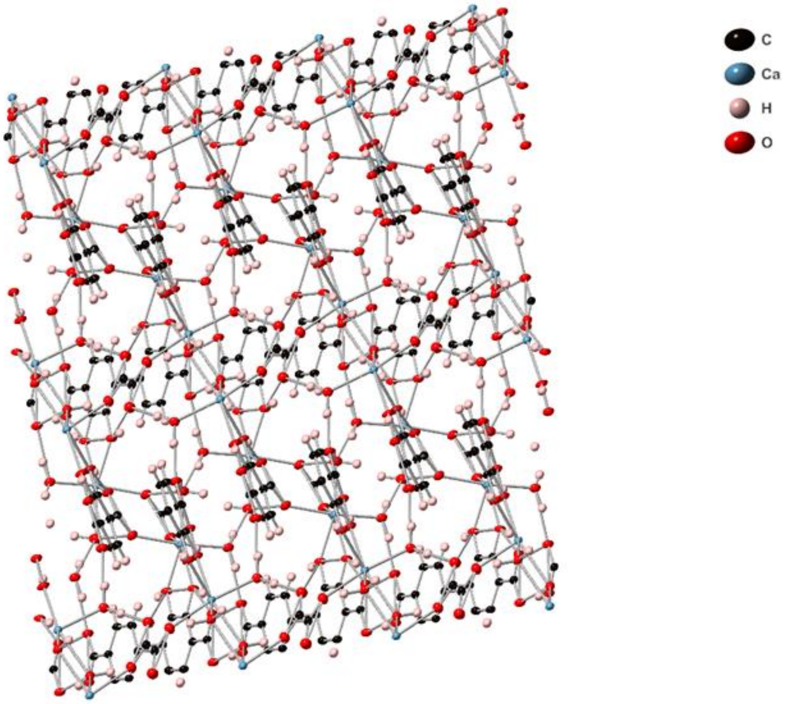

Figure S2: View of the 3D structure of SIMOF-4 (50 % probability ellipsoids) as seen down the crystallographic b-axis, and showing hydrogen bonds.

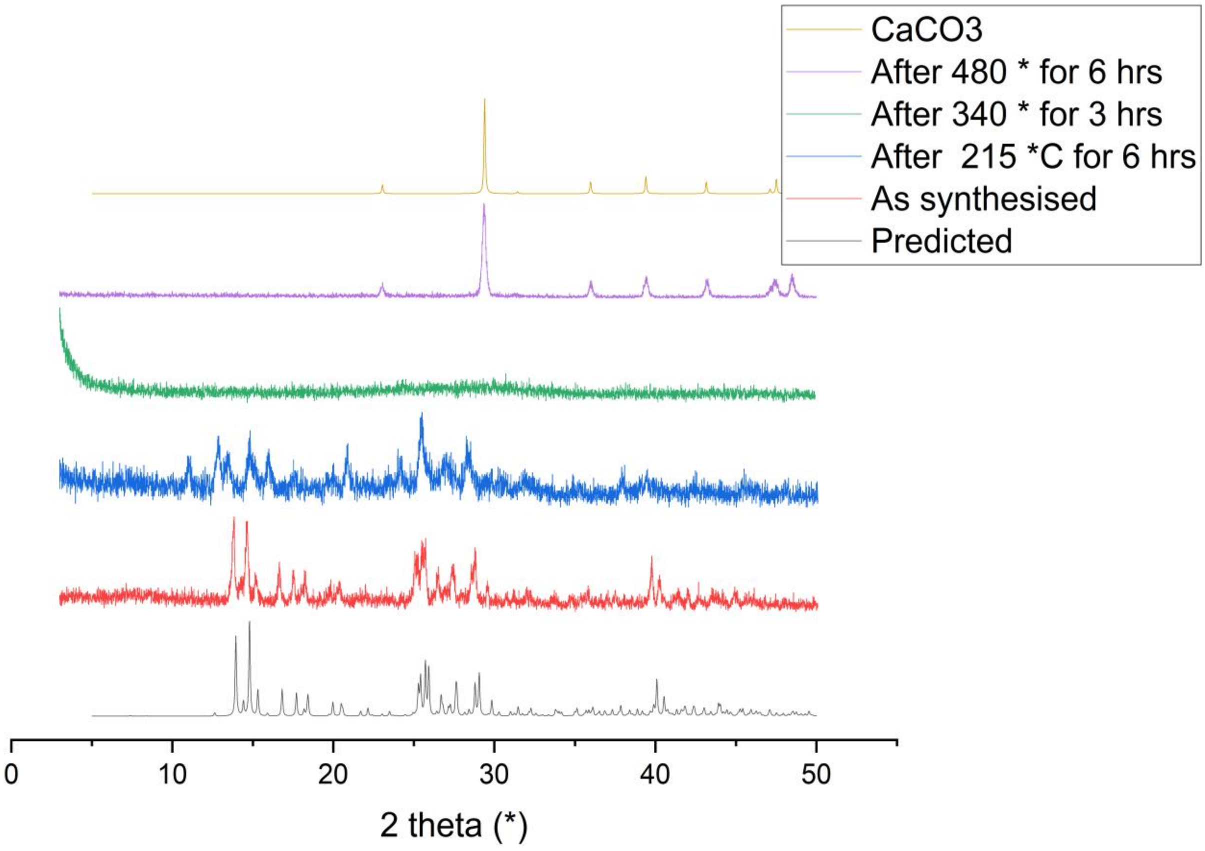

Figure S3: PXRD patterns of SIMOF-4 after different heat treatments, with simulated patterns of CaCO3 and SIMOF-4 for comparison. All obtained using Cu(Kα1) radiation.

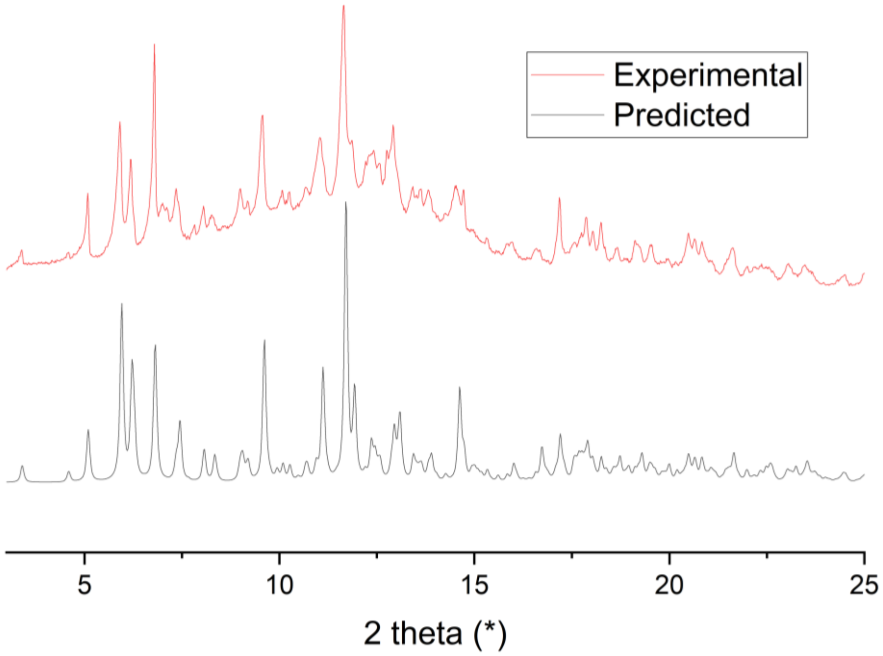

Figure S4: PXRD pattern of SIMOF-4-h simulated from scXRD structure compared with the experimental pattern. Obtained using Mo (Kα1) radiation.

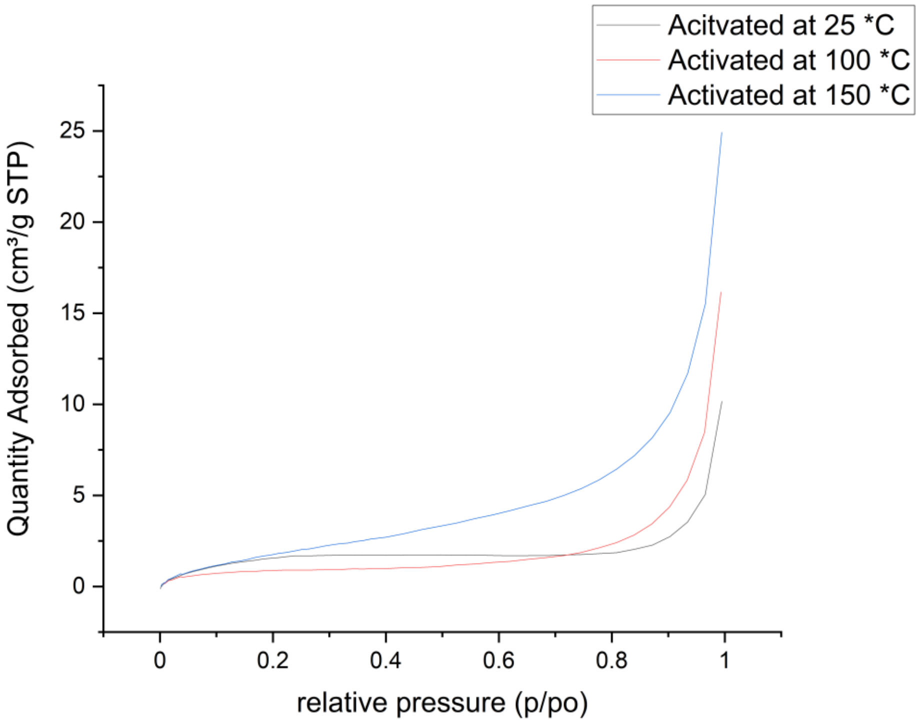

*FigureS5: N_2 Adsorbtion isotherms of SIMOF-4 after 3 different activation protocols of 25 *C, 100 *C and 150 *C all under vacuum.*

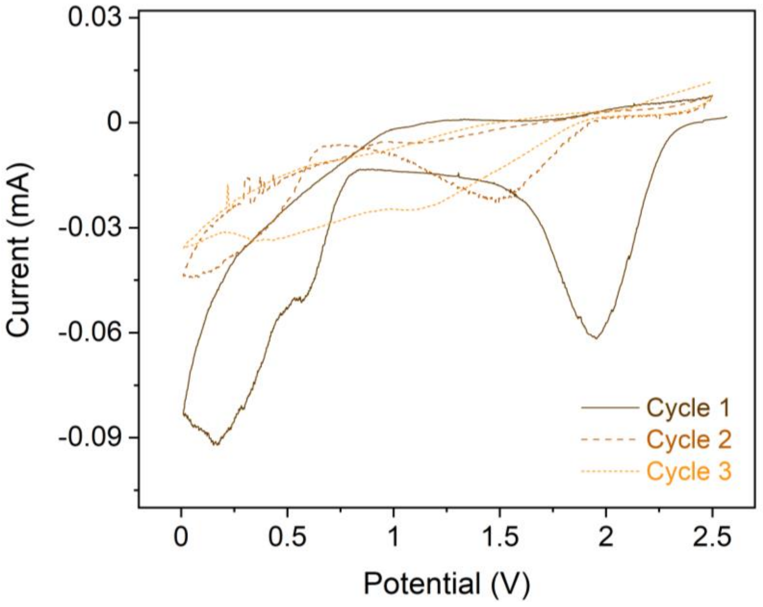

Figure S6: Cyclic voltammogram for SIMOF-4 recorded at a scan rate of 0.05 mV s^{-1} between 0.01-2.5 V.

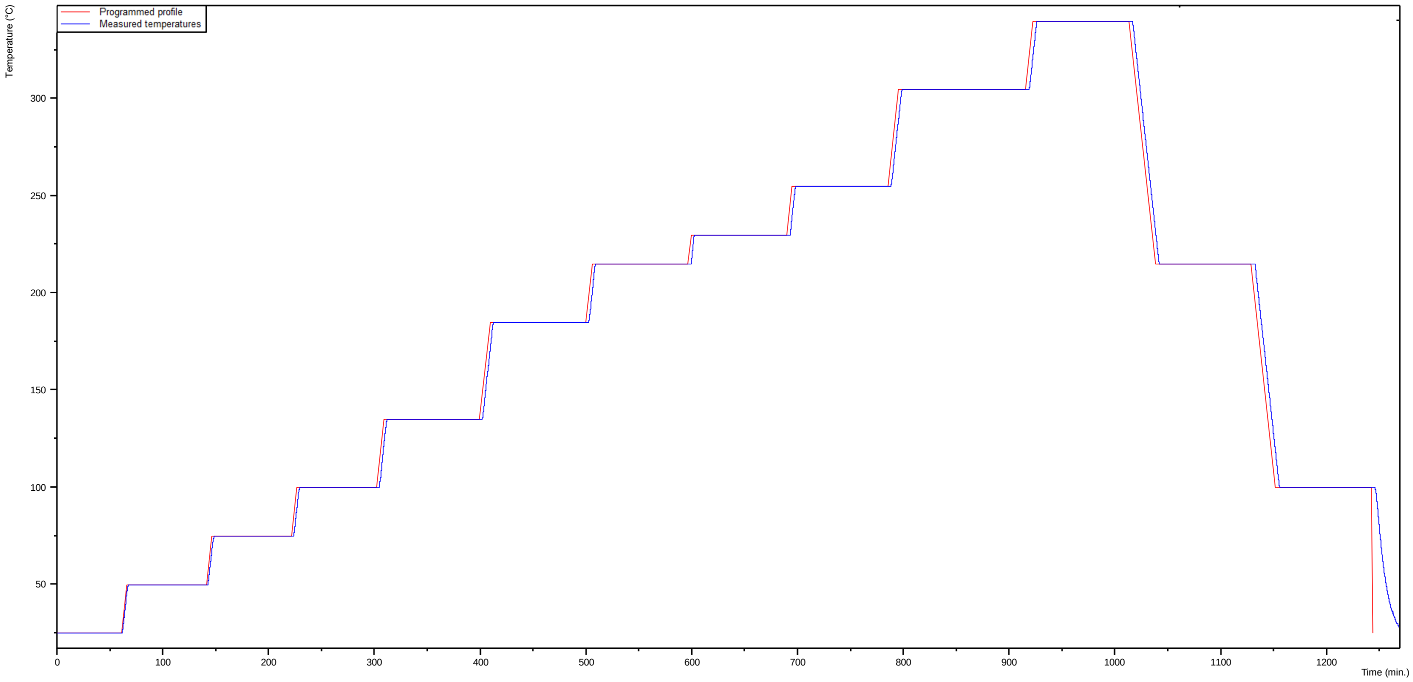

Figure S7: Temperature time profile for the VTXRD experiment performed on SIMOF-4, showing programmed and measured temperature profiles.
